# 1Menstruation: a possible independent health promoter, aging and COVID-19

**DOI:** 10.22088/cjim.13.0.155

**Published:** 2022

**Authors:** Alireza Bolourian, Jay Shen, Marjan Gharagozloo, Zahra Mojtahedi

**Affiliations:** 1 College of Pharmacy, Oregon State University, Corvallis, OR, USA; 2 Oregon Health and Science University, Portland, Oregon, USA; 3 Department of Health Care Administration and Policy, School of Public Health, University of Nevada, Las Vegas, NV 89154, USA; 4 Department of Neurology, Johns Hopkins University, Baltimore, Maryland, USA; 5 Autophgy Research Center, Shiraz University of Medical Sciences, Shiraz, Iran

**Keywords:** Aging, Gender, Menstruation, Women, Therapeutic plasma exchange

## Abstract

Women live longer than men. Cardiovascular disorders, cancers, and serious infectious conditions are less common among women than men. Recent data also indicate that women, particularly before menopause, are less susceptible to severe COVID-19, a viral infection hitting less-healthy individuals. The superiority of women regarding health has not been completely understood and partly been explained by estradiol beneficial effects on the microenvironment of the body, notably cytokine network. Estradiol cycles are aligned with menstruation cycles, a challenge for distinguishing their individual effects on human health. Large-scale, long-term studies indicate that hysterectomy, particularly at younger ages, is associated with an increased risk of mortality, cancer, or heart disorders. The underlying mechanisms for the increased risk in hysterectomized women are hard to be investigated in animal models since only a few primates menstruate. However, blood exchange models could resemble menstruation and provide some insight into possible beneficial effects of menstruation. Sera from animal models (neutral blood exchange) and also humans that have undergone therapeutic plasma exchange enhance the proliferation of progenitor cells in the culture and contain lower levels of proinflammatory factors. If menstruation resembles a blood exchange model, it can contribute to a healthier cytokine network in women. Consequently, menstruation, independently from estradiol health beneficial effects, can contribute to greater longevity and protection against certain disorders, e.g., COVID-19, in women. Investigation of COVID-19 rate/severity in hysterectomized women will provide insight into the possible beneficial effects of menstruation in COVID-19.

Women live longer than men (1-4). Worldwide, the average life span of women is found to be 4 to 5 years more than men (5). Women usually develop cardiovascular disorders at older ages compared to men and mostly after menopause (6). Overall, cancer and severe infectious disorders are less common among women compared to men (1-7). For instance, women have 40% less viral RNA than men in acute HIV infection and also a lower mortality rate in viral hepatitis (5). Recent data from the COVID-19 pandemic has been further documented that women can be less prone to severe infectious disorders (8, 9). The reasons why women are shielded against a wide range of illnesses have yet to be understood. Estradiol hormone has been mainly proposed to explain the superiority of women when it comes to health (8-15). Certain mechanisms for the protective effects of estrogen hormone have been suggested, notably regulating cytokine composition, the immune system, hypoxia-induced factor-1α signaling, and autophagy (5, 8-15). 

However, the differences in estrogen levels between genders can partly explain the better adaptation of women to life but there are still gaps that need to be filled by fresh theories. Menstruation, independent of estrogen, has yet to be acknowledged as a health promoter. However, there is evidence in humans indicating that menstruation and blood loss might exert health-promoting effects. Poorer health outcomes in women hysterectomized by age of 40, with or without oophorectomy, (16) cannot be justified by estradiol levels. Since only a few primates menstruate (17, 18), there is a paucity of animal models for more clarification of the role of menstruation, independent of estradiol effects. Blood exchange models might resemble frequent blood loss in menstruation. These models have been associated with a healthier cytokine network (19). We searched PubMed and Google Scholar for publications on hysterectomy and outcomes, COVID-19 and gender, and also blood exchange models, particularly those investigated longevity. We discuss here that menstruation, independent of estradiol effects, with possible similar mechanisms to blood exchange models (19) might contribute to a healthy microenvironment, longer lifespan in women, and health superiority against certain infectious disorders, such as COVID-19. 

Long-term adverse health outcomes in hysterectomized women

Long-term, large-scale studies indicate that hysterectomy, particularly at younger ages, is associated with an increased risk of mortality (20), cancer (16), and heart diseases (21-23). This association is mild and has not been consistent when the study is short-term, the sample size is small, or hysterectomy was performed at older ages (24-26). For example, Luoto et al. investigated the cancer rate among 25, 382 hysterectomized women and non-hysterectomized control women, registered in Finland Mass Screening Registry (20). Relative risk (RR) estimates of non-genital cancers among hysterectomized women were approximately 5% higher than in the non-hysterectomized cohort, particularly among women who had undergone total hysterectomy pre- or perimenopausally (20). Gierach et al. investigated 11, 247 women who had undergone hysterectomy with an average follow-up of 22.1 years (16). They found that total hysterectomy alone by age 40 was associated with increased mortality risk (HR40 years=1.08, 95% CI: 1.01, 1.15). The association was even stronger among patients who had undergone bilateral salpingo-oophorectomy as well (HR40 years=1.12, 95% CI: 1.04, 1.21) (16). However, Wilson et al. did not find a significant increase of all-cause death among hysterectomized women in a 21-year Australian population-based cohort study (25). The limitation of the study as stated by the authors was” that women were not asked about their age at hysterectomy until surveys 7 and 8 (in 2013 and 2016), so we could not explore whether the exact timing of surgery before the age of 50 years may vary the level of risk” (25). Iversen et al. investigated the long-term risk (mean > 20 years) of death from all causes among hysterectomized women. They reported that hysterectomy was not associated with a significantly altered risk of mortality regardless of age (24). However, the sample size of their study (3705 hysterectomized women) (24) was much smaller than studies by Luoto et al. and Gierach et al. who found contrasting results. (16, 20). Collectively, it seems that hysterectomy has a mildly negative effect on health that can only be discovered in a long-term, comprehensive study encompassing a large number of patients (16, 20). The underlying reasons for these negative associations have not been explained yet (16, 20). 

Blood-exchange models and a healthy cytokine network

Menstruation is a state of frequent blood loss, which might be partly resemble blood exchange models. Although the effects of menstruation on health have not been investigated due to the lack of reliable models, blood exchange models have been explored regarding their effects on health (19, 27-30). Certain blood exchange/removal models have long been acknowledged as health promoters (27), and recent research findings have uncovered some underlying mechanisms (19, 27-30). Comparative proteomic analysis on sera from humans underwent therapeutic plasma exchange (TPE) has revealed a molecular re-setting of the systemic signaling milieu. Factors promoting tissue maintenance, repair, and the immune response, such as interferon (IFN)-alpha/beta, thrombospondin-2, and C-X-C Motif Chemokine Receptor 2 (CXCR2) were increased in both humans and animal models (19). Interestingly, the serum pattern from the TPE model was consistent with those from “neutral blood exchange” (NBE) animal models. In these mice, half of the plasma was replaced with saline (19, 30). The replacement enhanced in-vivo muscle repair, reduced liver adiposity and fibrosis, and increased hippocampal neurogenesis (19, 30). In-vitro studies also revealed sera of the mice models exhibited functional rejuvenation effects, enhanced proliferation of progenitor cells in the culture, and induced rejuvenation of three germ layer tissues (muscle, liver, and brain-hippocampal neurogenesis) (19, 30). The TPE also mitigates viral diseases, notably influenza (19). Some reports also indicate that the TPE might have beneficial effects in the treatment of seriously ill COVID-19 patients (28, 31). Collectively, mounting evidence indicates that blood exchange can have beneficial health effects in both humans and animal models (19, 27-32).

Healthier cytokine network in Women: lessons from COVID-19

COVID-19 has been targeting people with unhealthy conditions including older ages, obesity, and chronic disorders (33-41). Recent worldwide experience with COVID-19 and less susceptibility of women for the severe disease has further brought attention toward the superiority of women regarding health (8, 9, 33, 37). Mounting evidence indicates that women have a more robust cellular and humoral immunity and cytokine network that have been partly attributed to the estradiol effects (8, 9, 14, 42). However, women were found not to be shielded against COVID-19 if the levels of proinflammatory cytokines such as interleukin-8 and interleukin-18 were high (14). 

Non-menopausal women have shown extra protection against COVID-19 compared to menopausal women (9). They also display different plasma levels of certain mediators, such as lowered levels of the key proinflammatory cytokines of interleukin-1β, interleukin-8, and T tumor necrosis factor-alpha (TNF-α) (9, 43-46). In other words, non-menopausal women have healthier autoregulatory proteins, which have been partly contributed to the protective effects of estradiol (43-46). Menstruation is the discharge of blood and mucosal tissue from the uterus through the vagina. A normal menstrual cycle occurs every 24 to 38 days, lasts 7 to 9 days, with 5 to 80 milliliters of blood loss until it stops by menopause (47, 48). Since menstruation, a frequent blood loss state, is aligned with the estrogen cycle, it is difficult to distinguish their individual effects on health. 

**Menstruation: a healthy cytokine network**

The association of hysterectomy with shorter life span and cancer (16, 20) indicates that mere frequent blood loss might have health-promoting effects, but there is a paucity of animal models (17, 18) to investigate the distinct effects of menstruation and mechanisms. Women, particularly before menopause, have a more robust cytokine network (9, 43-46), similar to blood exchange models (19, 30) and less susceptible to COVID-19 (9). Recent evidence indicates that blood exchange animal models have longer longevity, and their sera enhance the proliferation of progenitor cells in the culture and contain lower levels of proinflammatory cytokines (19, 30). Menstruation could resemble blood exchange models and by creating a healthier cytokine network, independent of estradiol effects, contribute to longer longevity and protection against COVID-19 in non-menopausal women. 

**Implications**

**Contribution of menstruation to health might further explain two other open questions:**

(i) Women live longer than men (1-4). We suggest that a healthier microenvironment created partly by menstruation can lead to the greater longevity of women compared to men.

(ii) COVID-19 disproportionately affects different groups of people because of different risk factors (42-40, 49, 50), such as menopause for female COVID-19 patients (9). Nonmenopausal women have milder severity and better outcome compared with male counterparts. Menopausal women also stay longer in hospital than nonmenopausal women (9). Several explanations have been proposed for the advantage of being a non-menopausal female against COVID-19, notably lower levels of pro-inflammatory molecules, such as complement component 3 (C3), interleukin-2R, interleukin-6, interleukin-8, and TNF-α in non-menopausal women (9). This healthier cytokine network has been explained by estradiol levels (9, 42-4^45^). We suggest that menstruation, similar to blood exchange models, by creating a healthier cytokine network might be another reason why women are less susceptible to COVID-19. As a consequence of health-promoting effects of hysterectomy, decisions on hysterectomy at younger ages should then be limited to malignant cases. Epidemiologic studies regarding COVID-19 susceptibility in hysterectomized women at young ages would clarify the extent that menstruation can contribute to protection against COVID-19. 

In conclusion, emerging evidence indicates that certain blood exchange models have been associated with the induction of rejuvenating factors and reduction of proinflammatory molecules. Non-menopausal women also have reduced levels of proinflammatory molecules which have been linked to estradiol effects. Hysterectomy at younger ages, which stops blood loss and possibly natural blood exchange, has been associated with long-term poor health outcomes. We gathered evidence that menstruation could resemble blood exchange models and contributes to a healthier cytokine network, greater longevity, and less susceptibility to COVID-19 in women compared to men. Whether hysterectomized women are more susceptible to COVID-19 needs more investigation. 
